# Epithelial-Mesenchymal Transition Proteins in Neuroendocrine Neoplasms: Differential Immunohistochemical Expression in Different Sites and Correlation with Clinico-Pathological Features

**DOI:** 10.3390/diagnostics10060351

**Published:** 2020-05-28

**Authors:** Elia Guadagno, Severo Campione, Sara Pignatiello, Giorgio Borrelli, Gianfranco De Dominicis, Nicolina De Rosa, Marialaura Del Basso De Caro

**Affiliations:** 1 Department of Advanced Biomedical Sciences, Pathology Section, Federico II University of Naples, Via Pansini 5, 80131 Naples, Italy; sarapignatiello91@libero.it (S.P.); giorgio.86@alice.it (G.B.); marialaura.delbasso@unina.it (M.D.B.D.C.); 2 Pathology Department, Cardarelli Hospital, Via Antonio Cardarelli 9, 80131 Napoli, Italy; severo@gmx.de (S.C.); gianfranco.dedominicis@aocardarelli.it (G.D.D.); 3 Pathology Department, Monaldi Hospital, Via Leonardo Bianchi, 80131 Naples, Italy; nicla.derosa@libero.it

**Keywords:** EMT, slug, twist, Ki67, neuroendocrine

## Abstract

The first step leading to metastasis, or for the acquisition of local invasiveness, involves changes in the phenotype of neoplastic cells in the primary tumor. The epithelial–mesenchymal transition (EMT) is a process that determines the acquisition of a form and a transcriptional program that are characteristic of mesenchymal cells, in epithelial cells. The factors involved in this process are E-cadherin and N-cadherin adhesion proteins and some transcription factors such as Slug and Twist. EMT is a site-specific mechanism that is also active in embryogenesis—embryonic cells are affected if invested in certain points, probably due to the signals emanating from the cells or groups of surrounding cells. It is known that neuroendocrine neoplasms have a biological behavior that differs in grading, staging, and site. The aim of our study was to investigate the immunohistochemical expression of EMT factors (Twist, Slug, and E-cadherin) in the neuroendocrine neoplasms of the gastrointestinal tract, the pancreas, and lungs, in 65 cases retrieved from the archives of the Department of Pathology, of three hospitals. The immunoscores were compared in each site and correlated with the clinico-pathological parameters. Statistical evaluation revealed an association between the higher Twist immunoscore and higher grading (*p* value < 0.0001) and staging (*p* value = 0.0055). Slug was detected only in pancreatic cases where its reduced expression was associated with a higher grading (*p* value = 0.0033). This data could be of diagnostic utility in the case of metastases from neuroendocrine neoplasm, to define the site of the primitive tumor when the traditional immunohistochemical panel is not sufficient. In summary, our results indicated, first that the EMT is also an active process in neuroendocrine neoplasms. To the best of our knowledge, this was the first study that evaluated the expression of EMT factors in neuroendocrine neoplasms of different districts.

## 1. Introduction

Neuroendocrine neoplasms (NEN) are rare (around 1–5 cases/100,000/year) and have a low incidence and high prevalence [1]. They arise from neuroendocrine cells that are disseminated all over the body and whose role is essential for the development of organs and the complex functional regulation of tissue. Their biology is strongly related to site, grading, and staging. The most common forms arise from the gastro–entero–pancreatic tract, specifically in the small intestine (19%), appendix (4%), and large intestine (20%), and their pathological classification relies on the morphological and proliferative markers (Table 1). In the gastroenteropancreatic tract [2], these neoplasms are categorized into well differentiated (neuroendocrine tumors, NET) and poorly differentiated forms (neuroendocrine carcinomas, NEC).

While NEC are high grade by definition, NET can be distinguished into low (G1), intermediate (G2), and high (G3) grade forms, on the basis of Ki67 [3] proliferative index/mitotic activity (Table 1).

More and more evidences [4] show that the G3 NET are distinct from G3 NEC, because the former are resistant to cisplatinum/etoposide therapy and show a higher overall survival than NEC. Mixed neuroendocrine/non-neuroendocrine neoplasms are labelled as MiNEN (Mixed Neuroendocrine Neoplasms), when each component constitutes ≥30% of the lesion.

In general, when compared to adenocarcinomatous lesions of the same site, neuroendocrine neoplasms show a better overall survival. However, among them, there are important site-specific differences [1,5] in terms of biology and overall survival, which could be explained by the extreme functional diversity of normal neuroendocrine cells, besides microenvironmental factors.

The current 2015 WHO Classification [6] of neuroendocrine neoplasms of the lungs and thorax catalogues four categories, on the basis of purely morphological parameters (cellular pleomorphism, absence/presence of necrosis, and mitotic activity) (Table 1). Although a grading system is not recommended in lung NEN, typical carcinoid, atypical carcinoid, and neuroendocrine carcinoma (large cell and small cell) are considered synonymous ^6^, respectively, with the G1, G2, and G3 forms.

Overall, differences among the districts are reflected into differences in the classification systems applied to each site. Despite this, however, a proposal to unify the nomenclature used for neuroendocrine neoplasms deriving from any district of the body is underway [7].

Tumorigenesis is a complex and dynamic process consisting of three steps—initiation, progression, and metastasis [8]. The first step for metastatic evolution is the acquisition of local invasiveness through major changes of cancer cell phenotype. Epithelial–mesenchymal transition (EMT) implies, in epithelial cells, the acquisition of a form and a transcriptional program that are characteristic of mesenchymal cells. This process is normally activated in embryogenesis and at the edge of a wound but also at the border of tumor infiltration. Every type of cancer can follow EMT process, before acquiring motility and invasiveness. Normal and pathological EMT [9] lead to alterations in the expression profiles of some genes—actually, the expression of epithelial markers (E-cadherin and cytokeratin) is repressed while there is an induction of vimentin and this phenotypic switch is turned on by the transcription factor (e.g., Slug, Snail, and Twist).

The clinical implications of EMT programs in cancer were extensively investigated through studies of immunohistochemical expression in several histotypes. For instance, in colo-rectal cancer [10], it was observed that a higher expression of Smad4, a linker of the TGFβ pathway, was related to higher levels of Snail, Slug, and Twist factors, as well as to a reduction of E-cadherin. In breast cancer, Slug is a negative prognostic factor [11] and, in a relevant study of metanalysis (including 2671 patients) [12], it was observed that a higher tissue expression of Twist was correlated with a greater tumor size, lymph nodes metastases, higher grading, and HER2 positivity.

A few studies were conducted to evaluate the expression of EMT factors implicated in gastro-intestinal and pancreatic neuroendocrine tumors [13,14,15]. No statistically significant correlation was detected between E-cadherin loss or Snail/Twist expression and overall survival, but an inverse correlation between E-cadherin and Snail/Twist was present. Recently, it was shown that in pancreatic NETs Slug-mediated EMT was driven by cancer stem cells [16] and that, in this context, immunohistochemical evaluation of Snail and E-cadherin was useful for predicting the risk of vessel invasion and metastasis [17]. Among pulmonary neuroendocrine neoplasms, most of the studies concerned small cell lung carcinoma and showed that EMT triggering can confer resistance to EGFR tyrosine kinase inhibitors [18].

The aim of our study was to assess the expression of EMT proteins in a collection of specimens of gastrointestinal, pancreatic, and pulmonary NEN, and to correlate it with clinico-pathological parameters.

## 2. Methods

### 2.1. Collective

The present study was multicentric and included 65 patients affected by neuroendocrine neoplasms, that underwent surgical resection at the Federico II University Hospital (38 cases), at the Monaldi Hospital (15 cases) and at the Cardarelli Hospital (12 cases) of Naples. The specimens were retrieved from the archives of the Department of Pathology of each hospital. The neoplasms were diagnosed in the period between 2001 and 2019, they were re-classified and graded according to the current World Health Organization (WHO) Classification system for neuroendocrine neoplasms of the gastroenteropancreatic tract (2019 WHO), and lung and thorax (2015 WHO). Staging was made in accordance with the American Joint Committee on Cancer (AJCC VIII edition) system [19].

### 2.2. Immunohistochemistry

Four micrometer sections were used for the immunohistochemistry. Sections were dewaxed in xylene, hydrated in a graded series of alcohol, and subjected to heat-induced antigen retrieval (10 mM Sodium Citrate, 0.05% Tween 20, pH 6.0). After blocking the endogenous peroxidase activity, the tissue was incubated with monoclonal antibody against Slug (*sc.166476* clone, mouse, SantaCruz, Santa Cruz, CA, USA- 1:100 dilution) or against E-cadherin (*EP700Y* clone, rabbit, Ventana, Oro Valley, AZ, USA- 1:100 dilution), or with the polyclonal antibody Twist (*49254*, rabbit, Abcam, Cambridge, United Kingdom- 1:500 dilution). For all antibodies, the incubation time was of 90 min. Subsequently, the slices were rinsed and incubated with the biotinylated secondary antibody, at room temperature, for 30 min. The bound antibody complexes were stained for 3–5 min, or until appropriate, for microscopic examination with diaminobenzidine, and they were then counterstained with hematoxylin (30 s) and mounted. Appropriate positive controls were chosen—normal breast tissue for Slug, glioblastoma for Twist, and breast cancer for E-cadherin. Negative control was obtained by omitting the primary antibody.

For all cases, Ki67 immunostaining was performed according to the automated procedure (Benchmark XT, Roche-Ventana, Oro Valley, AZ, USA).

### 2.3. Immunostaining Scoring System

The immunostaining of EMT proteins (Slug and Twist) was evaluated in tumor cells, in the stroma and in normal epithelium. The assessment of their immunostaining was based on the percentage of positive cells—no signal was set as “0”, ≤15% as “1”, >15% and <50% as “2”, and ≥50% as “3”.

For the E-cadherin staining, three categories were identified according to the percentage of cancer cells that preserved membranous staining: <5% positively stained cells were considered as ‘negative’ (score 1), 5–50% were classified as ‘reduced’ (score 2), and >50% stained cells as ‘preserved’ (score 3). All slices were reviewed by four experienced pathologists (M.D., E.G., S.C., and N.D.R.), using light microscopy. In discordant cases, the slides were re-evaluated on a multi-headed microscope to achieve consensus.

For the evaluation of the Ki67 Labeling Index, “hot spot” areas were chosen at a low magnification and an average of the values obtained on 5 adjacent fields (at least 500 neoplastic cells) was calculated [20]. When the score was discordant, it was assessed again, collegially, by manually counting the unlabeled and labeled nuclei on a camera-captured, printed image.

### 2.4. Statistical Evaluation

The study of association between the scoring group of each marker and clinico-pathological features (age, sex, site, grading and staging) was carried out by Fisher’s exact test. Spearman’s correlation test was used to examine the correlation between Twist and Ki67.

A *p* value ≤ 0.05 was considered statistically significant. All tests were two sided and carried out with the GraphPad Prism 5 software (GraphPad Software, La Jolla, CA, USA).

### 2.5. Ethical Approval

This was a retrospective study on tissue samples retrieved from the archives of three hospitals. The project was included in the POR CAMPANIA FESR 2014/2020 RARE.PLAT.NET project (CUP B63D18000380007) that was approved by the Ethics Committee of the Federico II University of Naples (2019/233, 16 July 2019).

For each patient, a written informed consent to use part of the specimen for scientific or the research scopes was provided.

### 2.6. Declarations

All procedures performed in the study, involving human participants, were in accordance with the ethical standards of the institutional board and with the 1964 Helsinki declaration, including signed informed consent for study participation.

## 3. Results

### 3.1. Clinico-Pathological Features

The collective (Table 2 and Table 3) consisted of 31 males and 34 females, aged between 27 and 79 years, with a mean age of 59.57 years and a median age of 61 years. They were all affected by the neuroendocrine neoplasms originating from the gastrointestinal tract, the pancreas, and the lung, respectively, in 28, 19, and 18 cases. The diagnosis was of NET G1 in 17 cases, NET G2 in 14 cases, TC in 10 cases, AC in 5 cases, NET G3 in 2 cases, and NEC in 17 cases. In two cases, NEC co-existed with adenocarcinoma, thereby, the final diagnosis was a high-grade Mixed Neuroendocrine Neoplasm (MiNEN). Ki67 L.I. ranged from 2% in NET G1 to 90% in NEC. We identified two staging groups—in 29 cases the disease was metastatic while in 36 it was non-metastatic (any pT and N0, M0).

Tissue was taken from the primary tumor in 60 cases and from a metastatic site in 5 cases (Table 2).

### 3.2. Differential Expression of Twist in Single Sites

**Figure 1:**
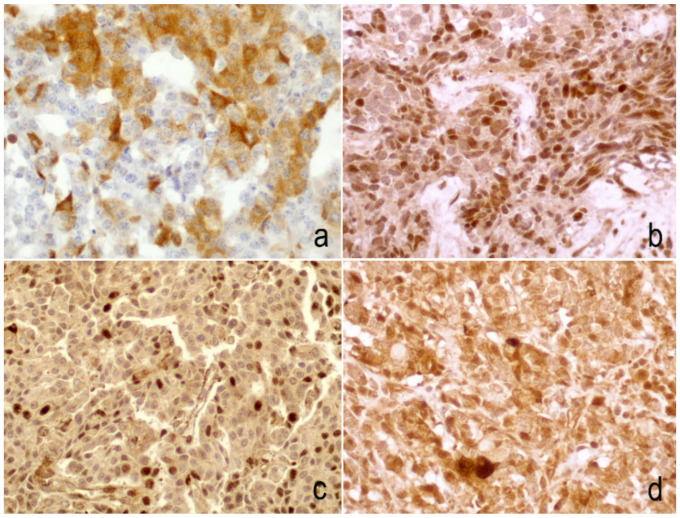
Examples of the Twist immunoscore—cytoplasmic signal was evident in 15–50% (**a**) and in >50% (**d**) of neoplastic cells; in some cases an exclusive nuclear signal was observed in >50% (**b**) and in 15–50% (**c**) of neoplastic cells. (**b–d**. 20× magnification; a. 40× magnification).

Twist protein showed a nuclear or cytoplasmic signal (Figure 1) and it was expressed in the neoplastic cells in 51 cases, with a score that was variable, being 1, 2, and 3 in 12, 19, and 20 cases, respectively. The cases were subsequently divided into two different scoring groups (independently from cytoplasmic or nuclear staining)—26 with absent/low signal (score 0–1) and 39 with moderate/high signal (score 2–3). Variable expression was also detected in normal tissue.

**Figure 2:**
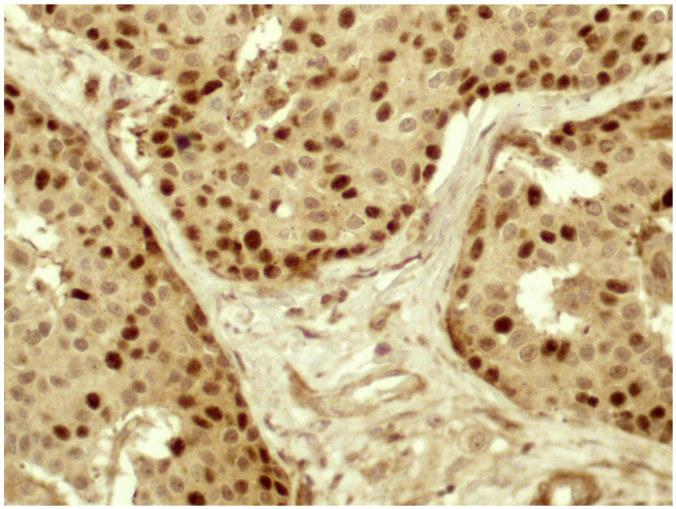
In most cases, Twist reactivity was located mainly at the periphery of the neoplastic nests, at strict contact with the stroma (40× magnification).

In most cases, especially in well-differentiated tumors, the signal was stronger at the periphery of neoplastic nests (Figure 2), where the epithelial cells were in contact with the surrounding stroma.

**Figure 3:**
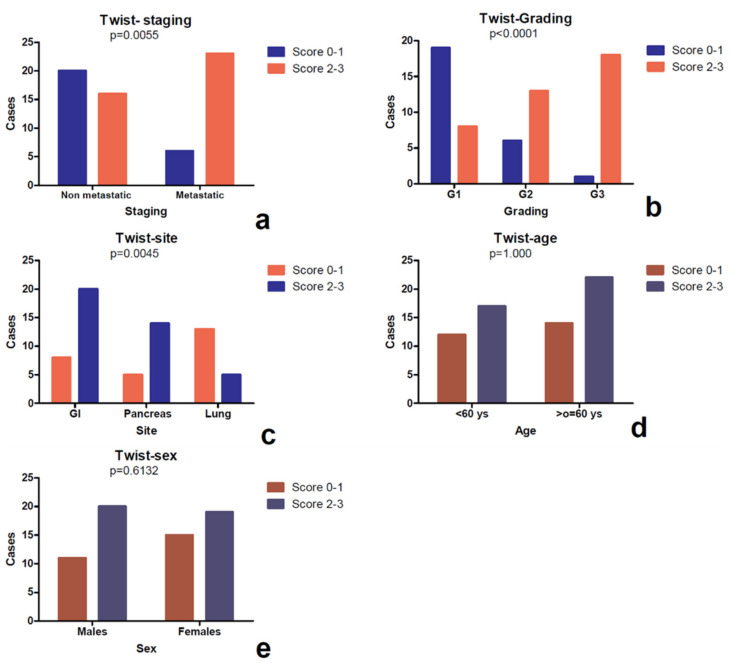
Study of association between the Twist score and the clinico-pathological parameters assessed by the Fisher’s exact test. It was observed that a higher signal (score 2–3) was more frequent in metastastic cases instead of non metastatic forms (**a**), in cases with higher grading (**b**) and in NEN located in the gastrointestinal tract, compared to pancreatic and pulmonary forms (**c**). No correlation was evident between Twist score and clinical parameters (sex and age) (**d**,**e**).

Fisher’s exact test did not reveal any statistically significant correlation with age and sex (Figure 3). However, a higher Twist expression was associated with a higher grading (*p* value < 0.0001), with metastatic staging (*p* value = 0.0055), and with gastro-intestinal tract localization (*p* value = 0.0045).

A separate statistical analysis for the cytoplasmic and nuclear stainings did not highlight relevant associations between immunoscoring and the clinico-pathological parameters.

In the subgroup of gastrointestinal and pulmonary neoplasms (Table 4), Twist was confirmed to be a factor related to higher grading (*p* value = 0.0034 and *p* value = 0.0129), but not with a higher staging.

A slight tendency, although not statistically significant, was observed between a higher Twist score and the metastatic stage (*p* value = 0.0638) in the gastrointestinal NEN subgroup.

No relevant association was observed between the Twist immunoscoring and the clinico-pathological parameters, among pancreatic lesions.

**Figure 4:**
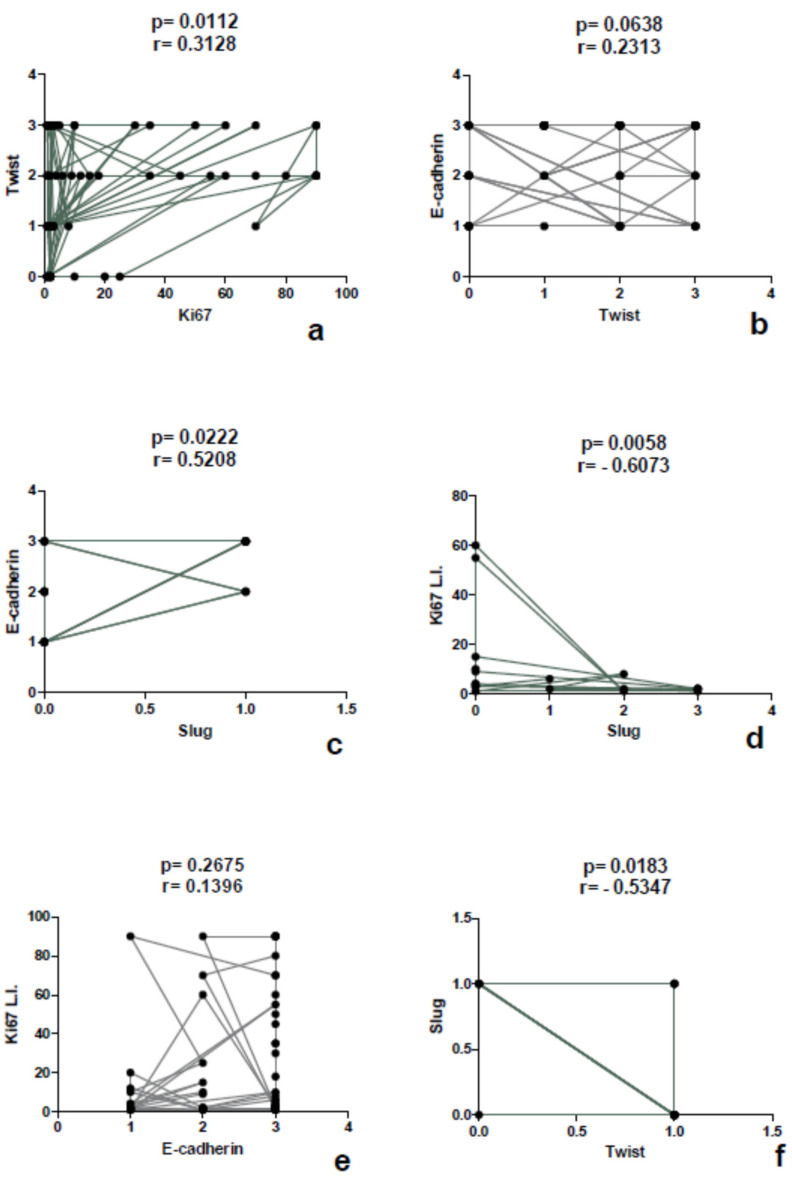
Correlation study between the markers performed by the Spearman’s exact test. Each dot represents a case.

The comparison between markers highlighted a direct correlation between Twist and Ki67 L.I. (*p* value = 0.0112, *r =* 0.3128) (Figure 4).

### 3.3. Expression of Slug

**Figure 5:**
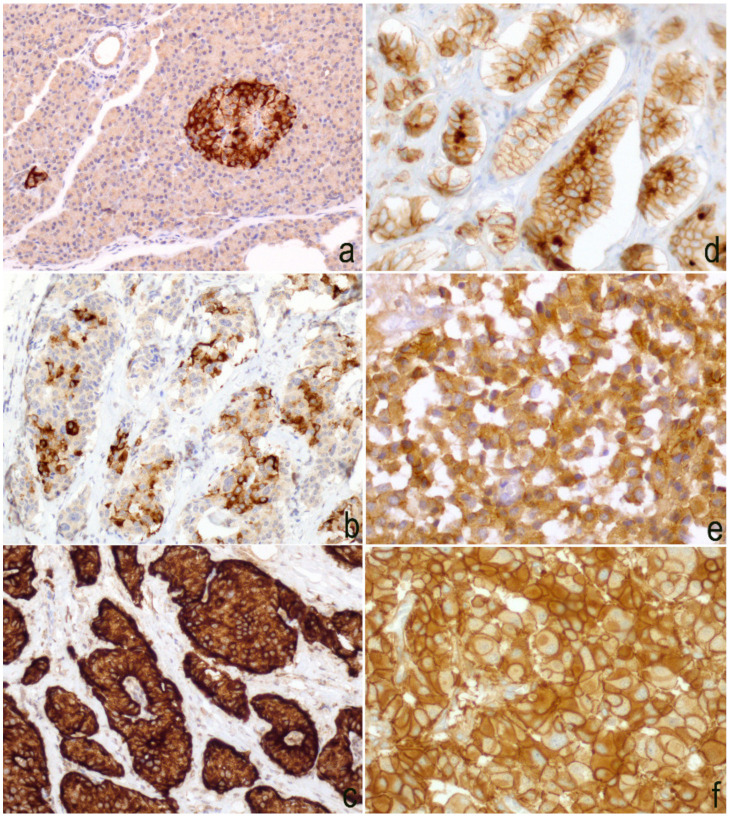
Slug immunoscore—(**a**) an intense cytoplasmic immunoreaction was evident in the normal pancreatic tissue that was located exclusively in the neuroendocrine compartment. Negative scores were exocrine pancreas (20× magnification); (**b**) cytoplasmic signal was observed in 15–50% of neoplastic cells (20× magnification); (**c**) in this field, strong and diffuse (>50%) immunoreaction was detected in neoplastic cells (40× magnification); (**d**) 20× magnification, and (**e**) 40× magnification. E-cadherin expression was reduced compared to cases where the expression was preserved (**f**) 40× magnification.

**Figure 6:**
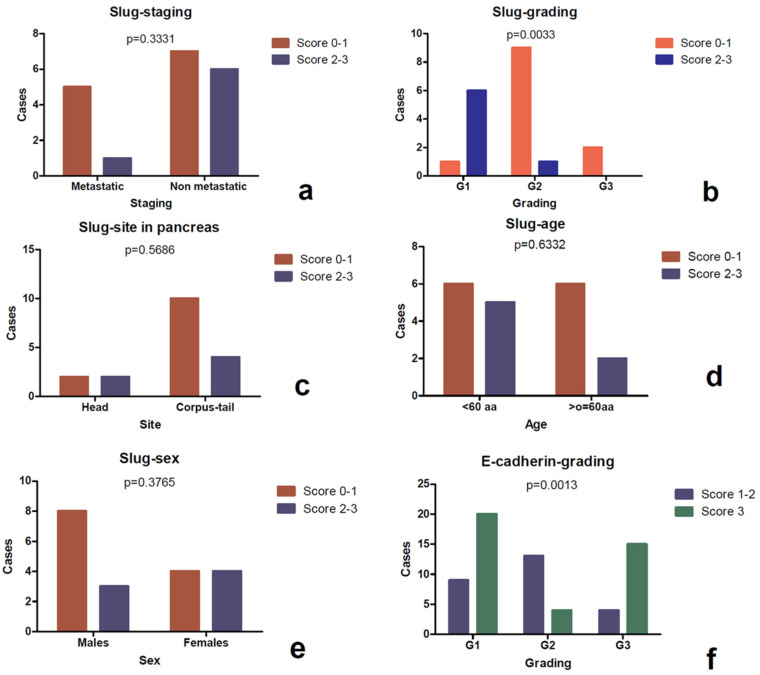
Study of association between the Slug score and the clinico-pathological parameters assessed by the Fisher’s exact test. It was observed that a higher Slug score (score 2–3) was correlated with a lower grading (**b**), as well as E-cadherin loss resulted was to be statistically correlated to higher grading (**f**). Slug was not associated with stageing (**a**), pancreatic regions (**c**), age (**d**) and sex (**e**).

Slug protein was expressed only in pancreatic neoplasms and was found to be homogeneously negative in all other locations. It showed cytoplasmic signal in 9 cases and the score was 1 in 2 cases, 2 in 3 cases, and 3 in 4 cases. Immunoreactivity was also observed in perilesional pancreatic tissue, but only in endocrine cells (Figure 5a–c). No association was detected between the immunoscore and the clinico-pathological parameters such as age, sex, site (head vs. corpus/tail), and stage. The most evident finding was a direct association between Slug lost/reduced expression and a higher grading (*p* value *=* 0.0033) (Figure 6), as well as an inverse correlation between Slug immunoscore and Ki67 L.I. (*p* value= 0.0058, *r*= −0.6073) and Twist (*p* value = 0.0030, *r* = −0.6428), using a Spearman’s exact test (Figure 4).

### 3.4. Expression of E-Cadherin

E-cadherin was observed at the intercellular junctions and with a partly reduced expression in the cytoplasm of some tumor cells (Figure 5d–f). The expression was preserved (score 3) in 39 cases, reduced (score 2) in 13 cases, and lost (score 1) in 13 cases. In the collective of 65 cases, a significant association was observed between E-cadherin reduced/lost expression and a higher grading (*p* value *=* 0.0013) (Figure 6f), and it was confirmed in the pancreatic (*p* value = 0.0116) and pulmonary (*p* value *=* 0.0017) subgroups (Table 4). Not confirmed in the subgroups was the statistically significant association observed in the whole cohort between the metastatic stage and E-cadherin reduction or loss of expression (*p* value = 0.001). No association was evident with the other clinico-pathological parameters. However, Spearman’s test highlighted a direct correlation (Figure 4) between the Slug and E-cadherin (*p* value = 0.0222, *r =* 0.5208) immunoscore.

## 4. Discussion

The aim of our study was to investigate the potential role of the Epithelial–Mesenchymal Transition process in neuroendocrine neoplasms, through an immunohistochemical study of Twist, Slug, and E-cadherin proteins. These factors were extensively studied in the oncological field [10,11,12], showing possible relevant prognostic factors.

As observed in the literature, both Slug and Twist showed heterogeneous expression in different tumor sites, as well as a variable association with clinico-pathological parameters [8]. Furthermore, within the same tumor, Twist expression appeared to be heterogeneous with a predilection, mainly in well-differentiated forms, at the periphery of epithelial nests, at strict contact with mesenchymal stroma.

In our collective, a higher expression of the Twist factor was observed in the cases with a higher grading. This finding could configure, for this marker, a prognostic role, as a discriminating factor in the post-operative follow-up among the cases that need more or less strict controls. Furthermore, Twist analysis could be a predictive factor for therapeutic options, representing both a useful criterion to choose more aggressive therapeutic regimens, and a therapy resistance factor, as demonstrated in gastric cancer, compared to regimens based on chemotherapy with Paclitaxel [21].

Tumors located in the gastrointestinal tract were characterized by a higher expression of Twist, compared to other sites.

EMT transcription factors are various and are normally expressed in different combinations in different cancer types, demonstrating that EMT programs differ cell by cell and neoplasm by neoplasm [8]. The acquisition of a mesenchymal phenotype can occur also without losing the pre-existent epithelial phenotype. Furthermore, each factor, alone, was not able to determine EMT changes. In our collective, Twist was more expressed in the gastro-intestinal tract, where, however, Slug was not expressed at all. This finding was in contrast with what was observed in other forms of cancer [10,11,12], as well as in neuroendocrine neoplasms of the pancreas where Slug was expressed.

Slug appeared to be a tissue-specific protein within neuroendocrine neoplasms and its loss of expression was associated with an increase in the histological grade of malignancy. This evidence could be of diagnostic utility in the case of metastases from neuroendocrine neoplasms, to define the site of the primitive tumor when the traditional immunohistochemical panel was not sufficient. Moreover, the evidence of Slug in the endocrine component of normal pancreatic tissue is suggestive of its possible role in embryogenesis.

A diagnostic utility of EMT factors could also be hypothesized in lung neoplasms. Indeed, it is well-known that the diagnosis of atypical carcinoid is based on the evaluation of morphological parameters (presence/absence of necrosis and mitotic activity), which are not very objective and have poor reproducibility [22]. The current classification system, in fact, is not very feasible and often does not reflect the actual biological behavior of the examined tumor. The finding of a greater Twist expression in the atypical carcinoid forms, with respect to the typical forms could, therefore, constitute a useful differential diagnostic factor, above all, in cases of limited and not representative biopsies of the whole tumor.

Surprising results were those obtained from the study of the E-cadherin expression. In accordance with what is known in the literature [10,11,23], the loss of expression of the adhesion protein was shown to be greater in cases showing a greater histological grade, therefore was more aggressive, even if no correlation with the tumor stage was observed. Opposite to what was observed in other tumors [10,11,12] was the direct correlation described between the E-cadherin and Slug expression. Generally [8], transcription factors act as an EMT inducer, hence they provoke the transformation from the epithelial cell phenotype into mesenchymal calls, starting from the loss of adhesion proteins (like E-cadherin) and the acquisition of new proteins (e.g., N-cadherin).

In summary, our results are the first to indicate that EMT is also an active process in neuroendocrine neoplasms. To the best of our knowledge, this is the first study that evaluate the expression of EMT factors in neuroendocrine neoplasms of different districts. The finding that Twist is more expressed in the gastro-enteric tract, together with the data, according to which Slug is expressed only in pancreatic NETs, is indicative of the real, possibly different nature and biology of these neuroendocrine neoplasms, according to the different onset site.

## Figures and Tables

**Table 1:** Classification of neuroendocrine neoplasms.

	GEP-NEN 2019 WHO		
Grade	Differentiation	Proliferation	Nomenclature
Low	Well differentiated	<2 mitoses/2 mm^2^ AND Ki67 < 3%	NET G1
Intermediate		2–20 mitoses/2 mm^2^ OR Ki67 3–20%	NET G2
High		>20 mitoses/2 mm^2^ OR Ki67 > 20%	NET G3
	Poorly differentiated(small cell or large cell)		NEC
	**Lung-NEN 2015 WHO**		
**Grade**	**Differentiation**	**Diagnostic Criteria**	**Nomenclature**
Low	Well differentiated	<2 mitoses/2 mm^2^ AND no necrosis	Typical carcinoid
Intermediate	Moderately differentiated	2–20 mitoses/2 mm^2^ OR foci of necrosis	Atypical carcinoid
High	Poorly differentiated (small cell or large cell)	>10 mitoses/2 mm^2^	- Small cell lung cancer - Large cell neuroendocrine cancer

**Table 2:** Clinico-pathological features of 64 study cases.

Cases	Sex	Age	Site	Diagnosis	Grading	Staging	Ki67	Twist	Slug	E-Cadherin
1	F	65	Vater papilla	NEC	G3	pT3N1Mx	50%	3	0	3
2	M	79	Ileum	NET	G1	pT3N0Mx	2%	1	0	3
3	M	56	Colon	NET	G2	pT3N1Mx	18%	2	0	3
4	F	59	Appendix	NET	G1	pT3N0Mx	1%	1	0	3
5	M	63	Colon	NEC	G3	pT3N1Mx	60%	3	0	3
6	M	54	Stomach	NET	G2	pT3N1Mx	5%	3	0	3
7	M	69	Colon	NEC	G3	pT4aN1Mx	35%	3	0	3
8	M	61	Ileum	NET	G1	pT4N1M1	1%	2	0	3
9	F	56	Stomach	NEC	G3	pT3N0M1	80%	2	0	3
10	F	43	Ombelical region °	NEC	G3	M1	70%	2	0	2
11	F	76	Ileum	NET	G1	pT4N1Mx	1%	2	0	3
12	F	50	Appendix	NET	G1	pT3N0Mx	1%	1	0	3
13	M	60	Ileum	NET	G1	(m)pT3N1Mx	1%	3	0	3
14	M	50	Stomach	NET	G3	pT4N1Mx	35%	2	0	3
15	F	74	Omentum °°	NEC	G3	M1	90%	2	0	3
16	M	53	Sigma	NEC	G3	pT3N0Mx	90%	2	0	3
17	M	71	Stomach	NEC	G3	pT4aN1Mx	45%	2	0	3
18	M	62	Rectum	NET	G2	pT1aNXMx	3%	3	0	3
19	M	61	Colon	NET	G1	pT4N1Mx	2%	1	0	3
20	F	64	Colon	NET	G2	(m)pT3N1Mx	3%	1	0	3
21	M	64	Colon	NEC(MiNEN)	G3	pT2N1Mx	70%	3	0	3
22	M	70	Colon	NET	G2	pT4 N1Mx	3%	1	0	3
23	F	66	Stomach	NET	G1	pT4N1Mx	2%	3	0	3
24	F	75	Appendix	NET	G1	pT1N0	2%	1	0	3
25	F	77	Jejunum	NEC	G3	pT4bN2Mx	90%	3	0	2
26	F	67	Rectum	NEC(MiNEN)	G3	pT3N1aMX	90%	2	0	3
27	F	52	Ileum	NET	G1	pT4N0M1	2%	1	0	3
28	M	55	Peritoneum °°°	NET	G3	M1	30%	3	0	3
29	M	59	Omentum ^#^	NET	G2	M1	3%	1	0	3
30	M	40	Pancreas (T)	NEC	G3	pT4aNxMx	60%	2	0	2
31	F	57	Pancreas (H)	NET	G1	pT1NxMx	1%	0	2	1
32	F	59	Pancreas (H)	NEC	G3	pT3N1Mx	55%	2	0	3
33	F	66	Pancreas (T)	NET	G2	pT1NxMx	4%	2	0	1
34	F	66	Pancreas (H)	NET	G2	pT2N0Mx	10%	3	0	2
35	F	27	Pancreas (T)	NET	G2	pT2NxMx	9%	2	0	2
36	F	62	Pancreas (H)	NET	G1	pT1N0Mx	2%	0	3	1
37	M	61	Pancreas (T)	NET	G2	pT3NxMx	15%	2	0	2
38	M	76	Pancreas (C)	NET	G2	pT3N0Mx	4%	3	0	1
39	M	52	Pancreas (T)	NET	G1	pT3N0Mx	1%	3	3	1
40	M	76	Pancreas (T)	NET	G1	pT3N1Mx	1%	3	0	3
41	F	53	Pancreas (C)	NET	G2	pT1N1Mx	8%	1	2	3
42	M	53	Pancreas (T)	NET	G1	pT1N1M1	2%	3	1	2
43	M	27	Pancreas (T)	NET	G1	pT2NXM0	2%	3	3	3
44	F	41	Pancreas (C)	NET	G1	pT1NXM0	2%	0	2	3
45	F	69	Pancreas (T)	NET	G1	pT1NXM0	2%	2	3	3
46	F	67	Pancreas (T)	NET	G2	pT3N0M0	3%	3	0	3
47	F	29	Pancreas (C)	NET	G2	(m)pT2N1MX	6%	2	1	3
48	F	62	Right lung (Inf)	AC	G2	pT1aN0Mx	1%	0	0	2
49	M	57	Right lung (Inf)	AC	G2	pT1aN0Mx	12%	2	0	1
50	M	74	Right lung (Sup)	TC	G1	pT1aN0Mx	1%	1	0	1
51	F	52	Right lung (Mid)	AC	G2	pT1aN0Mx	2%	0	0	1
52	F	76	Right lung (Mid)	TC	G1	pT2aN0Mx	20%	0	0	1
53	M	61	Right lung (Inf)	TC	G1	pT1bN0Mx	2%	0	0	2
54	F	63	Right lung (Inf)	AC	G2	pT2aN0Mx	10%	3	0	3
55	M	38	Left lung (Inf)	TC	G1	pT2aN0Mx	1%	0	0	1
56	F	68	Right lung (Inf)	TC	G1	pT1aN0Mx	1%	0	0	3
57	F	59	Left lung (Sup)	TC	G1	pT1aNxMx	2%	0	0	2
58	M	56	Right lung (Sup)	AC	G2	pT2aN0Mx	1%	3	0	2
59	F	37	Right lung (Inf)	TC	G1	pT2aN0Mx	2%	0	0	1
60	M	60	Left lung (Inf)	TC	G1	pT1bN0Mx	1%	0	0	2
61	F	73	Right lung (Inf)	TC	G1	pT1aN0Mx	10%	0	0	1
62	F	52	Right lung (Mid)	TC	G1	pT1aN0Mx	25%	0	0	2
63	F	53	Right lung (Inf)	NEC	G3	pT2bN0MX	90%	2	0	1
64	M	65	Right lung (Sup)	NEC	G3	pT1cN0MX	70%	1	0	3
65	M	74	Right laterocervical lymph node ^##^	NEC	G3	M1	90%	3	0	3

NEC (neuroendocrine carcinoma); NET (neuroendocrine tumor); MiNEN (Mixed Neuroendocrine Neoplasm); ° It was a metastasis from gastric NEC (G3); °° It was a metastasis from colonic NEC (G3); °°° It was a metastasis from colonic NET G3; # It was a metastasis from pancreatic NET G2; ## it was a metastasis from a pulmonary NEC; (T) pancreatic tail; (C) pancreatic corpus; (H) pancreatic head; Inf: inferior lobe; Mid.: middle lobe; Sup: superior lobe.

**Table 3:** Grouping of cases based on the clinico-pathological features.

Parameter	Measurement
Age (years)	
Mean	59.57
Median	61
Range	27–79
Sex	
Males	31 (48%)
Females	34 (52%)
Site	
Gastrointestinal	28 (43%)
Pancreatic	19 (29%)
Pulmonary	18 (28%)
Diagnosis	
NET	33 (51%)
TC	10 (15%)
AC	5 (8%)
NEC	17 (26%)
Grading	
G1	27 (42%)
G2	19 (29%)
G3	19 (29%)
Ki67 L.I.	
Range	1–90%
Mean	21.48%
Staging	
Metastatic	29 (45%)
Non metastatic	36 (55%)

**Table 4:** Statistical analyses using the Fisher’s exact test.

	Twist					
	Gastrointestinal		Pancreatic		Pulmonary	
	Score 0–1	Score 2–3	Score 0–1	Score 2–3	Score 0–1	Score 2–3
Age						
<60 years	3	7	4	7	5	3
≥60 years	5	13	1	7	8	3
*p* value	1.000		0.3378		1.000	
Sex						
Males	3	12	3	4	5	4
Females	5	8	2	10	8	1
*p* value	0.4097		0.3047		0.2941	
Staging						
Metastatic	5	17	5	1	13	4
Non metastatic	4	2	7	6	0	1
*p* value	0.0638		0.3331		0.2778	
Grading						
G1	6	4	3	5	10	0
G2	2	2	2	7	2	3
G3	0	14	0	2	1	2
*p* value	**0.0034**		0.3852		**0.0129**	
Slug						
Score 0–1	--	--	1	4	--	--
Score 2–3	--	--	11	3	--	--
*p* value	--		**0.0379**		--	
	**E-Cadherin**					
	**Gastrointestinal**		**Pancreatic**		**Pulmonary**	
	**Score 1–2**	**Score 3**	**Score 1–2**	**Score 3**	**Score 1–2**	**Score 3**
Age						
<60 years	1	9	6	5	7	1
≥60 years	1	17	5	3	7	3
*p* value	1.000		1.000		0.5882	
Sex						
Males	0	15	5	2	6	3
Females	2	11	6	6	8	1
*p* value	0.2063		0.6332		0.5765	
Staging						
Metastastic	2	15	1	5	8	9
Non metastastic	0	1	7	6	0	1
*p* value	1.000		0.1770		1.000	
Grading						
G1	0	10	1	6	9	1
G2	0	4	8	2	5	0
G3	2	12	2	0	0	3
*p* value	0.3406		**0.0116**		**0.0017**	
